# Managing Ethical Dilemmas in End-Stage Neurodegenerative Diseases

**DOI:** 10.3390/geriatrics2010008

**Published:** 2017-01-20

**Authors:** James Alvin Low, Esther Ho

**Affiliations:** 1 Department of Geriatric Medicine and Clinical Ethics Committee, Khoo Teck Puat Hospital, 90 Yishun Central, Singapore 768828, Singapore; 2 Department of Geriatric Medicine, Tan Tock Seng Hospital, 11 Jalan Tan Tock Seng, Singapore 308433, Singapore; esther.ho@mohh.com.sg

**Keywords:** dementia, Parkinson’s, bioethics, advanced, end-of-life, Advance Care Planning, capacity, autonomy

## Abstract

Neurodegenerative diseases are chronic, progressive and incurable illnesses that ultimately lead to death. The patient deteriorates inexorably towards the terminal phase of the disease when he becomes mentally and physically incapacitated. This article discusses the many ethical and moral dilemmas faced by the clinician and family members as they care for patients with neurodegenerative illnesses approaching the end of life. Topics discussed will include steps on how to assess mental capacity and decision-making capability, advance care planning, withholding and/or withdrawing treatment, food refusal, the do-not-resuscitate order and euthanasia. An approach to ethical decision-making incorporating Jonsen’s 4-topic approach will also be discussed briefly.

## 1. Introduction

Neurodegenerative diseases share a common pathological etiology that is marked by progressive neuronal cell loss which is postulated to be caused by neurovascular changes, changes in blood–brain barrier permeability and cerebrospinal fluid flow, the deposition of neurotoxic substances such as amyloid protein, oxidative stresses and genetic predisposition [1,2,3]. Among the conditions that fall under this category of illnesses are Alzheimer’s Dementia (AD), Parkinson’s Disease, Motor Neurone Disease, Spinocerebellar Ataxia, Huntington’s Disease and other rarer conditions. These diseases are characterized by their progressive, incurable and incapacitating nature, and are marked by a gradual and relentless deterioration in the patient’s physical and, in many cases, cognitive function over time. An euphemism for AD, the ‘long goodbye’, could apply equally to all neurodegenerative conditions as, left untreated, these diseases progress to cause the loss of a patient’s executive function and mental capacity, leading ultimately to death. It robs patients of their identity and autonomy, erodes individual independence, thus making them dependent on others for their activities of daily living and causing them to lose their higher cognitive functions. Numerous ethical issues emerge and evolve in relation to the stage and severity of these disorders. From a whole person perspective, these diseases take a toll on the patients’ psychological and emotional wellbeing, adversely impact their families and impair their overall quality of life. From a bioethical perspective, these diseases cause the patients to lose their autonomy and decisional-making capacity. Autonomy and the right to self-determination form one of the pillars of Medical Ethics and underpin the decision-making process of managing most, if not, all conditions in medicine. This article will use AD and PD as references while traversing the various stages of neurodegenerative diseases and highlighting the relevant ethical red flags to contemplate and consider along the way. We will also discuss Jonsen’s 4-topic approach to tackling ethical dilemmas in the later stages of these diseases [4].

## 2. Current Ethical Issues in Neurodegenerative Disorders

The diagnosis of a neurodegenerative condition signifies a life event that changes the patient’s world completely. To many, it is viewed as a calamity that heralds the start of a long, progressive course towards the loss of the person’s independence, dignity and, eventually, life itself. Neurodegenerative diseases exact a heavy burden and toll on patients, families, caregivers as well as society. They contribute to increased healthcare cost and give rise to numerous ethical and moral dilemmas, especially towards the end of life [5]. We now look at the various ethical dilemmas that may arise at the different stages of the diseases, paying particular attention toAD and PD, which epitomize this group of illnesses.

### 2.1. Early Stage

As alluded to earlier, the diagnosis of a neurodegenerative disease has many ramifications. There is a stigma attached to it, one that denotes progressive weakening, frailty, dependency and finally death. In many instances, there is a tendency, either by families or healthcare workers, to hide the diagnosis from the patient on the pretext of protecting the latter from being harmed by bad news and the assumption that the patient would not be able to grasp the gravity of the condition anyway. When carers conspire with the healthcare team to hide a diagnosis from the patient, the act of collusion would have occured. This practice, prevalent in the Eastern cultures, deprives the patient of his autonomy and right to self-determination. It degrades the patient’s personhood and autonomy. Barring rare exceptions, the truth, regardless of how painful it may be, has to be revealed to the patient if the patient asks for it. The physician owes a duty of care to the patient and has to remain faithful and loyal to the patient whatever the circumstances may be. In addition, the duty of the physician does not end with the disclosure but continues beyond, to support and counsel the patient and family as necessary. Medical confidentiality should also be observed to protect the privacy of the patient and safeguard him from discrimination and stigmatization. Society has a role to play in accepting people with neurodegenerative illnesses and in ensuring that the latter is treated with dignity, respect and equitability, especially towards the later stages of the illnesses when community care assumes an important role.

With advancements in the areas of genetic testing and testing for biomarkers of the preclinical stages of neurodegenerative illnesses such as Alzheimer’s and Huntington’s dementia, it is possible to detect these conditions early and allow for timely treatment to be instituted [6]. However, these tests pose serious ethical dilemmas for the individual who, although not yet a patient but having been identified as high risk for developing the illness, is likely to be discriminated against in terms of his employment opportunities and insurance coverage [7,8,9]. There may be scant protection from discrimination for such ‘patients’ on top of the fact that they will have to face life with much anxiety, worry and the prospect of developing full blown symptoms of a disease which is incurable later. Apart from a research imperative, one could question the utility of screening for such conditions in the first instance. However, it has been shown that predictive testing may generally bring more benefits than risks to individuals with preclinical neurodegenerative conditions [10].

Moreover, individuals who have relatives with neurodegenerative conditions may still wish to seek clarity on their status for peace of mind. Informed consent must be obtained to carry out such tests and the approach taken when disclosing positive results of genetic and biomarker testing, should be one that is conducted with sensitivity, empathy and in strict confidence [11,12,13].

Invariably, there is a gradual loss of mental capacity and decisional-making ability as the patient’s cognition and executive function deteriorate. The prerequisites for a person to exercise his autonomy are intact mental capacity, voluntariness and being adequately informed of the risks and benefits of the intervention or treatment to be considered. The loss of any of these would put the autonomy of the patient in question. It would therefore be in the interest of persons with neurodegenerative diseases to plan ahead in anticipation of this eventuality by making an advance medical directive (AMD), advance care plan (ACP) or lasting power of attorney (LPA), to predetermine end-of-life healthcare, financial and general welfare matters, respectively [14,15,16,17]. These measures help the patient exercise his or her autonomy when mental capacity and decisional-making ability are lost later in life. ACP which addresses end-of-life healthcare issues should be carried out while the patient still retains mental capacity. It is a process that involves making informed decisions about end-of-life care and appointing a substitute decision maker who will help make decisions on behalf of the patient using substitute judgment and the best interest principle for the patient, who no longer is able to make such decisions on his own. It is usually conducted by trained facilitators who guide the discussions with the patient and the nominated substitute decision maker, exploring the former’s values, beliefs, wishes and choices [18]. Advance care planning is geared for the future and is regarded as anticipatory care for those with incurable and progressive diseases. It maintains the individual’s autonomy even when mental capacity is loss, via the expression and documentation of one’s decisions and wishes through the Physician Order Life-Sustaining Treatment (POLST) form and the Substitute Decision Maker [19].

### 2.2. Intermediate Stage

As the disease progresses, the patient gradually loses various modalities of his function, requiring varying levels of assistance, initially in his instrumental activities of daily living and subsequently, the basic activities of daily living. For the dementias, there is loss of immediate-, short- and intermediate-term memory. In addition, there may be worsening motor deficits leading to falls and accidents, speech and swallowing difficulties and mood disorders. All these act to impair the ability of the patient to communicate clearly and coherently, deleteriously affecting the patient’s mental capacity and autonomy. At this stage, it may be necessary to assess mental capacity for those who missed the opportunity earlier to make their ACP or LPA. Numerous jurisdictions have enacted laws governing the assessment of mental capacity and the processes surrounding its application. There are statutes governing the making of an LPA and appointing a deputy (for those unable to make their own LPA arising from loss of mental capacity) [20,21,22]. The laws also seek to protect the interests of persons lacking mental capacity through the office of the Public Guardian which oversees the enforcement, implementation and execution of the aforementioned enactments.

The importance of mental capacity looms large in the management of neurodegenerative diseases, especially those that present with dementia. It is important to ascertain mental capacity in ethical decision-making, particularly when deciding whether or not to activate a patient’s AMD, ACP or LPA. We will discuss later, how this applies in ethical decision-making, especially towards the end of life. At this stage it is imperative that strict assessment guidelines are adhered to before declaring someone as having lost mental capacity (and hence his autonomy). This paves the way for treatment to be decided by the healthcare team and substitute decision maker, based on the best interest principle and substitute judgment (especially if no ACP, AMD or LPA had been made) [23].

The ACP and AMD processes adopt a 2-stage approach to help the patient exercise his or her autonomy for future healthcare. The first stage is when the patient makes the advanced decisions while possessing mental capacity. Usually, a trained facilitator explores with the patient, in the presence of the SDM, the former’s values, beliefs and wishes with regard to future medical treatment and care. The second stage is activated only when and if, the patient loses mental capacity (when the ACP and AMD come into force). As long as the patient retains mental capacity and even if he should be in the last stages of life, he should be consulted directly for his decisions, regardless of whether there is an ACP or AMD document in place. 

It is evident that the act of ascertaining mental capacity is of the utmost importance in any ACP, AMD or LPA programme. The usual practice of assessing mental capacity includes the following steps:

- the patient understands the information presented, e.g., the treatment or intervention being considered
- the patient is able to register, retain, remember and recall the information given
- the patient is able to weigh the risks and benefits of a having as well as not having the treatment
- the patient is consistent and able to communicate his decisions to the healthcare team

The act of assessing mental capacity is task specific as understanding is predicated largely upon the complexity of a treatment or procedure, and time specific alluding to the fact that mental capacity may fluctuate between lucidity and confusion, especially during an acute illness. The onus is on the assessor to ensure that all conditions conducive for exercising mental capacity are in place and that the patient fully understands the procedure or treatment being offered at a time when the latter is lucid. A patient is presumed to have mental capacity unless proven otherwise and an apparently unwise decision does not necessarily mean that a patient lacks mental capacity.

### 2.3. Late Stage 

Some of the most difficult ethical dilemmas present themselves in the later stages of neurodegenerative illnesses, when the patient becomes severely incapacitated psychologically and physically. Those with dementia may present with behavioral and psychological symptoms that imposes a great burden on the caregivers. Such patients may end up in long-term care facilities, where a host of unique bioethical issues could arise.

At this stage, one has to grapple with issues related to the extent of care of patients who are dependent, frail and are deemed to have poor quality of life. A considered approach to treatment and care would require that the risk-burden versus benefit ratio is weighed thoroughly in order to obviate the use of disproportionate, burdensome and futile treatment in this vulnerable group. Dilemmas related to withholding or withdrawing treatment near the end of life are some of the most common reasons patients are referred to hospital ethics committees [24]. While withholding treatment is an omission and withdrawing treatment an act, the former passive while the latter active, both are morally similar, that is, one does not carry a heavier weight of culpability with either an omission or a commission.

The concept of medical futility is closely related to the act of foregoing medical treatment. Schneiderman et al have defined medical futility in terms of quantitative and qualitative futility [25,26]. Quantitative medical futility refers to a treatment where the probability of it benefitting the patient is so small or miniscule that it would be unjustifiable to carry it out when weighed against the harm and burden that it imposes on the patient. Qualitative futility on the other hand refers to a treatment effect that does not provide any benefit to the patient in a meaningful way, especially when the resultant effect is poor quality of life. An example for this would be sustaining the physiological functions of the body with life support systems without any chance whatsoever of recovery in consciousness or awareness of the patient.

For most patients with end-stage neurodegenerative conditions, cardiopulmonary resuscitation and other life sustaining measures, may appear futile and should be weighed judiciously in accordance with patients’ prior wishes [27]. When the goal of treatment is supportive and for comfort measures, a palliative care approach would be the best recourse for these patients, who are often in the terminal stages of their illness.

Neurodegenerative illnesses may be complicated by behavioral and psychological symptoms towards the later stages. This exerts a heavy toll on the family especially if the patient is agitated, violent and disruptive. The use of neuroleptics in such circumstances should be a measure of last resort. It is known that neuroleptics, which act as chemical restraints bring with them complications such as strokes, movement disorders, instability, falls, etc. Restraint use, whether chemical or physical, robs the patient of his autonomy and dignity. They bring harm (maleficience) to the patient by causing immobility and, as such, predispose the patient to pressure ulcers, infections, dehydration, injuries and a host of other problems.

The only justification, if at all, for using restraints is when the patient is in danger of hurting or harming himself, or those around him and where the benefits far outweigh the potential risk or harm. If the only recourse is to resort to such restrictive measures, then it is imperative that the patient is monitored very closely for any of the aforementioned complications and the goal should be to discontinue the measure as soon possible [28].

There are occasions when a patient refuses to take his or her medications and the temptation is to ‘conceal’ the medication in the former’s food and drinks, alluding to the practice of covert medication use [29,30]. Similar to the use of restraints, this practice goes against the patient’s autonomy, is deceptive and undermines the principles of fidelity and truthfulness. Covert medication use should only be carried out in exceptional cases when the benefits of treatment far outweighs the risks/burden and, even then, be carried out judiciously and reviewed with the intention to discontinue the practice as soon as permissible [31].

Food refusal and dysphagia are common late complications of neurodegenerative conditions and are vexing ethical dilemmas for clinicians caring for the elderly. The decision on whether to insert a feeding tube or provide other artificial means of nutritional support is a complex one and will require a multidimensional approach to decision-making. An example of such an approach would be the Jonsen’s 4-topic approach to resolving ethical dilemmas in the clinical context, which is discussed in the next section [4].

## 3. Jonsen’s 4-Topic Approach to Resolving Ethical Dilemmas

This is a practical approach and framework used by many hospital ethics committees to resolve clinical ethical dilemmas. It categorizes the information, facts and details to be considered and weighed into four main themes or topics, described as quadrants- Medical Indications, Patient Preferences, Quality of Life and Contextual Features. Each of these ‘topic’ is equal in weightage, i.e., none takes precedence over the others. We now briefly describe the four topics in greater detail.

### 3.1. Medical Indication 

The physician must consider the disease and its natural history, whether it is reversible with treatment and the prognosis of the disease if left untreated. One also weighs the risk–benefit ratio of providing versus withholding a treatment or intervention, taking into account, the probability of success, the complication rates, etc. The physician is expected to adhere to the principles of beneficience and non-maleficience, act in the patient’s best interest and fulfill his fiduciary duties to the patient.

### 3.2. Patient Preference

This serves to recognize the patient’s autonomy and right to self-determination. It honours and respects the patient’s wishes as far as possible but within limits. For situations where there is lack of mental capacity, as in patients with neurodegenerative conditions, the ACP and AMD would apply, and the substitute decision maker could help in the decision-making process.

### 3.3. Quality of Life

The merits of a treatment or intervention cannot be measured solely with a quantitative matrix. One should also consider how it will impact the present and future quality of life of the patient. What would be the opportunity cost of prolonging life temporally and temporarily but at a great cost, burden and suffering to a patient who does not have long to live anyway? What does ‘quality of life’ mean to a patient at different stages of the illness and of his life? Quality of life seen from the various dimensions of the human is complex, unique to each individual and a subjective phenomenon that may not be adequately understood even with substitute judgment of family members.

### 3.4. Contextual Features

The complexity of clinical ethical dilemmas is exacerbated by social, cultural, religious, ethnic and legal considerations. Health economics and organizational practices also have a bearing on the decision-making process. Utilitarianism and distributive justice are some of the ethical principles that are applied in this quadrant of the window.

The above framework affords a cogent and systematic approach to solving clinical ethical dilemmas that crop up frequently in the care and management of patients with advanced neurodegenerative conditions. 

## 4. Conclusions

Patients with neurodegenerative disease are a frail and vulnerable group that deserves every care and consideration when receiving treatment throughout the healthcare continuum. Care should be person-centered, holistic, multidisciplinary and ethically sound from the time of diagnosis right till the time of death. The ultimate goal would be to respect the patient’s autonomy, to act in the best interest of the patient and honour the patient–doctor relationship.
